# PERCEIVED DISCRIMINATION, ANGER CONTROL, AND INFLAMMATION IN MIDLIFE: THE ROLE OF EARLY-LIFE ADVERSITY

**DOI:** 10.1093/geroni/igz038.3045

**Published:** 2019-11-08

**Authors:** Chioun Lee, Jennifer Coons, Lexi Harari

**Affiliations:** 1 University of California-Riverside, Riverside, California, United States; 2 California State University, Fullerton, California, United States

## Abstract

Early life adversity has severe consequences for adult biological health particularly in minority group individuals. Two ways in which it may be possible to reduce these negative consequences on adult health are individual differences in perceived discrimination due to early life adversities and learning internal skills (i.e. anger control) to help cope with early life adversities and perceived discrimination. The current study utilized 2,118 participants (55% female) from the MIDUS Projects. Early life adversities included three constructs: low socioeconomic status, family instability, and abuse (sexual, physical, and emotional). The best-fitting model from the latent class analyses revealed four distinct groups: 1) no early life adversities group, 2) low socioeconomic status only group, 3) low socioeconomic status and family instability group, and 4) all three early life adversities group. Minority groups were more likely to reside in the all three early life adversities group. Perceived discrimination was measured via two pathways: lifetime discrimination and daily discrimination. Anger control (one measure of an internal skill one learns to cope with early life adversities and perceived discrimination) was assessed with an anger-control scale. Inflammation markers were used as an indicator of biological health. Experiencing more early life adversities was related to greater inflammation and this relationship was partially explained via the pathway of greater early life adversities, greater perceived lifetime/daily discrimination, worse anger control, and greater inflammation. The findings support the need for a more holistic measure of early life adversities as it has a clear impact on adult inflammation.

